# *Cryptococcus neoformans* and *Cryptococcus gattii* clinical isolates from Thailand display diverse phenotypic interactions with macrophages

**DOI:** 10.1080/21505594.2018.1556150

**Published:** 2018-12-06

**Authors:** Adithap Hansakon, Putthiphak Mutthakalin, Popchai Ngamskulrungroj, Methee Chayakulkeeree, Pornpimon Angkasekwinai

**Affiliations:** a Department of Medical Technology, Faculty of Allied Health Sciences, Thammasat University, Pathumthani, Thailand; b Graduate Program in Biomedical Science, Faculty of Allied Health Sciences, Thammasat University, Pathumthani, Thailand; c Department of Microbiology, Faculty of Medicine, Siriraj Hospital, Mahidol University, Bangkok, Thailand; d Division of Infectious Diseases and Tropical Medicine, Department of Medicine, Faculty of Medicine Siriraj Hospital, Mahidol University, Bangkok, Thailand

**Keywords:** *Cryptococcus neoformans*, *Cryptococcus gattii*, macrophages, immune responses, fungal uptake

## Abstract

*Cryptococcus*-macrophage interaction is crucial in the development of cryptococcocal diseases. *C. neoformans* and *C. gattii* are major pathogenic species that occupy different niches and cause different clinical manifestations. However, the differences of macrophage interaction among these species in affecting different disease outcomes and immune responses have not been clearly addressed. Here, we examined the macrophage uptake rates, intracellular loads and intracellular proliferation rates of *C. neoformans* and *C. gattii* clinical isolates from Thailand and analyzed the effect of those interactions on fungal burdens and host immune responses. *C. neoformans* isolates showed a higher phagocytosis rate but lower intracellular proliferation rate than *C. gattii*. Indeed, the high intracellular proliferation rate of *C. gattii* isolates did not influence the fungal burdens in lungs and brains of infected mice, whereas infection with high-uptake *C. neoformans* isolates resulted in significantly higher brain burdens that associated with reduced survival rate. Interestingly, alveolar macrophages of mice infected with high-uptake *C. neoformans* isolates showed distinct patterns of alternatively activated macrophage (M2) gene expressions with higher *Arg1, Fizz1, Il13* and lower *Nos2, Ifng, Il6, Tnfa, Mcp1, csf2* and *Ip10* transcripts. Corresponding to this finding, infection with high-uptake *C. neoformans* resulted in enhanced arginase enzyme activity, elevated IL-4 and IL-13 and lowered IL-17 in the bronchoalveolar lavage. Thus, our data suggest that the macrophage interaction with *C. neoformans* and *C. gattii* may affect different disease outcomes and the high phagocytosis rates of *C. neoformans* influence the induction of type-2 immune responses that support fungal dissemination and disease progression.

**Abbreviation:** Arg1: Arginase 1; BAL: Bronchoalveolar lavage; CCL17: Chemokine (C-C motif) ligand 17; CNS: Central nervous system; CSF: Cerebrospinal fluid; Csf2: Colony-stimulating factor 2; Fizz1: Found in inflammatory zone 1; HIV: Human immunodeficiency virus; ICL: Intracellular cryptococcal load; Ifng: Interferon gamma; Ip10: IFN-g-inducible protein 10; IPR: Intracellular proliferation rate; Mcp1: Monocyte chemoattractant protein 1; Nos2: Nitric oxide synthase 2; PBS: Phosphate-Buffered Saline; Th: T helper cell; Tnfa: Tumor necrosis factor alpha.

## Introduction

Cryptococcal disease is caused by fungal infection with two major pathogenic species, *C. neoformans* and *C. gattii*. Infection by these fungi is acquired by inhaling desiccated yeasts or basidiospores that result in pulmonary or disseminated cryptococcosis. Diseases caused by *C. neoformans* infection are often associated with immunocompromised hosts, while *C. gattii* infection has been reported to cause disease in immunocompetent individuals. In patients with immunosuppressed conditions, particularly HIV-positive patients, cryptococcal meningitis occurring by *C. neoformans* infection is one of the most common life-threatening diseases that have been documented. In contrast, patients infected with *C. gattii* often suffer from respiratory illness in addition to having meningoencephalitis [1,2]. The differences in primary target organs and patterns of lung and brain pathology resulting from the infection of these two species have been revealed [3,4]. Indeed, the divergence in the host immune responses to these species may determine disease outcomes and be responsible for the differences in disease pathogenesis of *C. neoformans* and *C. gatii* infections.

Studies in humans and murine cryptococcosis models have emphasized the importance of T helper cell-mediated immunity in controlling cryptococcal infection [5–7]. Infection with *C. gattii* was found to modulate host immune response by subverting the induction of protective Th1 and Th17 cells [8]. During a chronic infection, *C. neoformans* elicited Th2 type-2 immune responses, including the induction of IL-4, IL-5 and IL-13 that provided a permissive environment for fungal proliferation and dissemination [9,10]. In addition to adaptive immunity, the collaborative function of innate immune response is required to stimulate fungal clearance. It has been suggested that alveolar macrophage and phagocytosis may play crucial roles in early control or exacerbation of cryptococcal infection [11–13]. Indeed, macrophages may alter their phenotypes to classical (M1) or alternatively activated (M2) macrophages in exposure to type-1 or type-2 cytokine environments, respectively. While M1 macrophages play a role in the early containment of *C. neoformans*, M2 macrophages promote fungal persistence [14]. Recently, *C. neoformans* with high phagocytosis rate by macrophages was reported to be correlated with CNS fungal burden and poor clinical outcomes [15]. Moreover, *C. gattii* strains from the Vancouver Island outbreak were found to exhibit the increased intracellular proliferative capability in macrophages that contribute to the virulence in murine cryptococcosis model [16].

Although many studies have emphasized the roles of fungal uptake and proliferation in macrophages as critical virulence factors in cryptococcal disease, their involvement in the virulence of *C. neoformans* and *C. gattii* and the effect of this interaction on the induction of immune responses remain unclear. In this study, we showed that the phagocytosis rate of *C. neoformans* was higher than that of *C. gattii*, while intracellular proliferation rate of *C. gattii* was greater than that of *C. neoformans*. High fungal uptake of *C. neoformans* but not the intracellular proliferation rate of *C. gattii* was associated with the fungal loads in a mouse model of cryptococcosis. Interestingly, mice infected with high-uptake *C. neoformans* strains displayed enhanced M2 macrophages and type-2 immune responses. Our studies suggest that the initial phagocytosis of *C. neoformans* but not *C. gattii* by macrophages may determine poor clinical outcomes by modulating M2 macrophages and eliciting the unfavorable type-2 immune response.

## Materials and methods

### Mice

BALB/c mice, female, 6 to 8 weeks old, were obtained from Nomura Siam International Co., Ltd, Thailand. Mice were housed under specific pathogen-free conditions in the animal facility of Thammasat University. At the end of the experiment, mice were euthanized by controlled gradual displacement with CO_2_ using a flow meter in accordance with IACUC and the American Veterinary Medicine Association (AMVA) guidelines on euthanasia. All animal studies were approved by the Thammasat University Animal Care and Use Committee (008/2557).

### *Cryptococcus* strain

All clinical isolates of *Cryptococcus* spp. (23 isolates of *C. neoformans* and 18 isolates of *C. gattii*) were obtained from the Department of Microbiology, Faculty of Medicine, Siriraj Hospital, Mahidol University. All the isolates were identified with the RapID™ Yeast Plus System (Thermo Fisher Scientific, MA, USA). Urease and melanin production test were also used to classify *C. neoformans*/*C. gattii* species. L-Canavanine–glycine–bromothymol blue (CGB) agar was used to differentiate *C. neoformans* from *C. gattii* [17]. The reference strains of *C. neoformans* (H99) and *C. gattii* (R265) were used as control strains in all experiments. *Cryptococcus* strains were stored in 20% glycerol at −80°C until use. *Cryptococcus* was thawed and maintained in Sabouraud dextrose agar (SDA) (Thermo Fisher Scientific) at room temperature. A single colony of yeast cells was resuspended in Sabouraud dextrose broth (SDB) and cultured at 37°C then shaking at 200 rpm for 24 hours before *in vitro* and *in vivo* infection [8,15].

### Macrophage culture

The murine macrophage cell line, J774 (ATCC® TIB-67^TM^) was cultured as previously described [15]. Briefly, macrophages were cultured in complete Dulbecco’s Modified Eagle Medium (cDMEM), DMEM medium supplemented with 10% heat-inactivated fetal bovine serum (FBS), 1% L-glutamine and 1% penicillin and streptomycin (Invitrogen) in a 5% CO_2_ humidified atmosphere at 37°C. Before infection, 1.5 × 10^5^ cells of J774 macrophages were cultured in 24-well culture plate (Corning) containing cDMEM for 24 hours at 37°C with 5% CO_2_.

### In vitro phagocytosis and intracellular proliferation assay

The rate of phagocytosis (uptake) and intracellular proliferation (IPR) by macrophages of *Cryptococcus* spp. were determined as previously described [15]. Briefly, J774 macrophages were cultured in serum-free medium for 2 hours followed by activating with 1 µg/ml phorbol myristate acetate (PMA) (Sigma-Aldrich) for 30 minutes at 37°C with 5% CO_2_ before infection. Cryptococci washed with 1x Dulbecco’s Phosphate-Buffered Saline (DPBS) (Invitrogen) were then opsonized with 1 µg/ml of anti-capsule mAb 18B7 (kindly provided by Arturo Casadevall, Albert Einstein College of Medicine, New York, USA). For *in vitro* infection, macrophages were exposed to opsonized cryptococci at 1:10 ratio and incubated at 37°C with 5% CO_2._ Cryptococcal uptake was determined by the number of intracellular cryptococci within the macrophage 2 hours after infection. After 2-hour infection, extracellular cryptococci were removed, macrophage cultures were lysed with sterile distilled water and intracellular cryptococci were counted using a hemocytometer and confirmed by plating on Sabouraud dextrose agar (SDA) (Thermo Fisher Scientific) for the analysis of CFU. To determine the intracellular cryptococci (ICL; intracellular cryptococcal load), the remaining wells were maintained and lysed at 18 and 24 hours as described above. The IPR of *Cryptococcus* at 18 and 24 hours postinfection was calculated from the number of intracellular cryptococci at 18 or 24 hours postinfection divided by the number of intracellular cryptococci at 2 hours postinfection.

### Murine model of *Cryptococcus* infection

For *in vivo* infection, the 24-hour cryptococcal cultures were washed by PBS, counted using a hemocytometer, and resuspended in PBS at a concentration of 1 × 10^6^ yeast cells/ml as previously described [8]. After anesthetization with isoflurane, mice were treated with PBS or infected with *Cryptococcus* by intranasal inoculation of 50 µl of the yeast cell suspension (5.0 x 10^4^ yeast cells/mouse). For survival analysis, BALB/c mice were infected with the high-uptake and the low-uptake *C. neoformans* strains as described above (8 mice/strain) and monitored for their survival by inspection twice daily for 60 d and euthanized if they appeared to be in pain or moribund [8].

### Organ isolation and CFU assay

Following euthanasia, the lungs and brains of *Cryptococcus*-infected mice were excised, weighed and homogenized in sterile PBS with 1% penicillin and streptomycin using a tissue homogenizer and plated at various dilutions on SDA (Thermo Fisher Scientific). CFU was calculated following incubation at 30°C for 48 hours.

### Bronchoalveolar lavage collection

At the indicated time points, an incision was made below the jaw to expose the trachea. A 22-gauge catheter was inserted in the airway and secured in place by a string. A total of 0.5 ml ice-cold PBS containing with 5 mM EDTA was instilled through the catheter and the lung was subsequently washed 5 times. The bronchoalveolar lavage (BAL) fluid was collected after centrifugation at 3,000 rpm for 5 min, and the supernatants (BAL fluids) were stored at −80°C for cytokine analysis and arginase activity.

### Alveolar macrophage isolation

Alveolar macrophages were recovered from bronchoalveolar lavage cells. Cells were washed and cultured in serum-free medium at 37°C, 5% CO_2_ for 2 hours in a 24-well plate. After incubating for 2 hours, nonadherent cells were removed and alveolar macrophages (adherent cells) were washed and harvested with a rubber scraper for further RNA extraction. Cell viability was determined using trypan blue exclusion and purity was analyzed with CD11b staining by FACS analysis [18].

### Quantitative RT-PCR

Alveolar macrophage RNA was extracted using TRIzol® reagent according to the manufacturer’s instructions. RNA was served as a template for cDNA synthesis using oligo-dT, random hexamers and MMLV reverse transcriptase (Invitrogen). To quantify macrophage polarization markers, cDNA samples were amplified using iTaq^TM^ universal SYRB® Green Supermix (Biorad Laboratories). Expression levels of target genes were normalized to endogenous actin (*Actb*) transcript levels, and relative quantification of samples was compared with PBS control serving as the baseline.

### Antigen-specific cytokine production

Lung draining lymph nodes harvested from PBS-treated and *Cryptococcus*-infected mice were minced in RPMI medium supplemented with 10% heat-inactivated fetal bovine serum (FBS), 1% L-glutamine, and 1% penicillin and streptomycin through a 100 μm sterile nylon mesh to prepare cell suspension solution. Single cell suspensions were plated in a 24-well plate and stimulated with live or heat-killed *Cryptococcus* cells (at a ratio of 2:1 *Cryptococcus* cells to leukocytes). Following 72 hour stimulation at 37°C with 5% CO_2_, the culture supernatant was collected and kept at −80°C for cytokine analysis [8].

### Cytokines measurement

BAL fluids and lung draining lymph node culture supernatants were used to determine the concentration of cytokines. IL-4, IFN-γ and IL-17 were analyzed using the antibody pairs from BD Pharmingen and IL-13 was assessed using ELISA kits obtained from R&D Systems. All cytokine assays were performed according to the manufacturer instructions.

### Arginase activity assay

The activity of arginase enzyme was determined as previous described [19]. Briefly, 100 µg/ml BAL fluids were added to the activation solution (50 mmol Tris-HCl and 10 mmol MnCl_2_, pH 7.5). After incubating at 56°C for 10 minutes, 0.5 M L-arginine (pH 9.7) was added to the activated mixture, incubated at 37°C for 2 hours, followed by adding acid stop solution (H_2_SO_4_:H_3_PO_4_:H_2_O, 1:3:7). For the colorimetric determination of urea, 9% α-isonitrosopropiophenone (ISPF) (Sigma-Aldrich) in ethanol was added, and the mixture was heated at 100°C for 45 min. Urea concentration was determined spectrophotometrically by measuring absorbance at 490 nm. The activity of arginase enzyme was determined as a concentration of produced urea (µg/ml).

### Statistical analysis

Each experiment was performed two to three times. Data are presented as mean value ± SD. Data were analyzed using unpaired t test (2-tailed) as well as the one-way ANOVA with Turkey’s post hoc analysis. Correlation study was analyzed using Spearman’s rank correlation coefficient. Survival curves were evaluated for statistical significance with Kaplan-Meier survival curves, and P values were obtained from a log-rank test. Statistical analysis was performed with GraphPad Prism 5 Software. A value of p <0.05 was considered significant.

## Results

### Differences in phagocytosis rate and intracellular proliferation rate of *C. neoforman*s and *C. gattii* by macrophages

**Figure 1.**
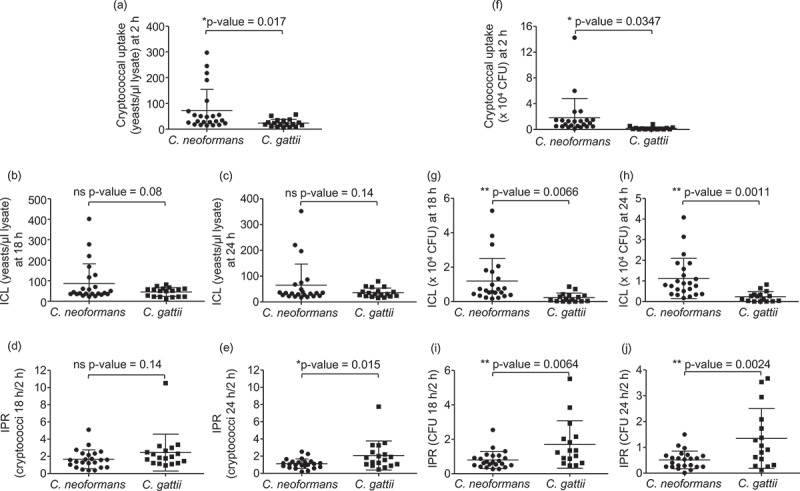
Phagocytic uptake, intracellular load and proliferation rate of *C. neoformans* and *C. gatii* clinical isolates by macrophages. The J774 macrophage was infected by clinical isolates of *C. neoformans* and *C gattii* at a 1:10 ratio followed by lysis at 2, 18 and 24 h postinfection to determine cryptococcal uptake at 2 h, intracellular loads (ICL) at 18 h and 24 h and intracellular proliferation rate (IPR) at 18 h and 24 h by (a – e) counting with hemocytometer and (f-j) enumerating using CFU assay. Each dot represents one clinical isolate. Results were expressed as the mean of 3 to 6 experimental repeats. Error bars denote mean ± SD. Significance was determined using unpaired t test (2-tailed) * p <0.05, ** p <0.01, ns = not significantly different.

Differences between the interaction of macrophages with *C. neoformans* and *C. gattii* have not been clearly investigated. Therefore, to determine the effect of *C. neoformans* and *C. gattii* infection on host macrophages, we first compared the phagocytosis rate of 23 isolates of *C. neoformans* and 18 isolates of *C. gattii* using the established murine macrophage–like cell line J774 [16]. By counting cells using a hemocytometer or enumerating the colony forming unit (CFU), the phagocytic uptake of *C. neoformans* was significantly greater than those of *C. gattii* (Figure 1(a), P = 0.017 and Figure 1(f), P = 0.0347, respectively). We next evaluated whether *C. neoformans* and *C. gattii* may differ in the intracellular fungal load and intracellular proliferation rate because they have been known to be associated with the virulence of *C. neoformans* and *C. gattii*, respectively. Although we observed no difference between the intracellular load of *C. neoformans* and *C. gattii* by counting the number of intracellular cryptococci at 18 or 24 hours (Figure 1(b,c)), the intracellular load determined as CFU of *C. neoformans* was higher than those of *C. gattii* (Figure 1(g), P = 0.0066 and Figure 1(h) = 0.0011). However, we found that the intracellular proliferation rate at 18 and 24 hours was higher in macrophages infected with *C. gattii* than those infected with *C. neoformans* (Figure 1(d), P = 0.14, Figure 1(e), P = 0.015, Figure 1(i), P = 0.0064 and Figure 1(j), P = 0.0024). These data suggest that although *C. neoformans* is more readily engulfed by macrophages than *C. gattii, C. neoformans* is able to proliferate in macrophages less than *C. gattii*.

### The high uptake rate of *C. neoforman*s but not high intracellular proliferation rate of *C. gattii* correlated with the cryptococcal virulence in a murine model of cryptococcosis

**Figure 2.**
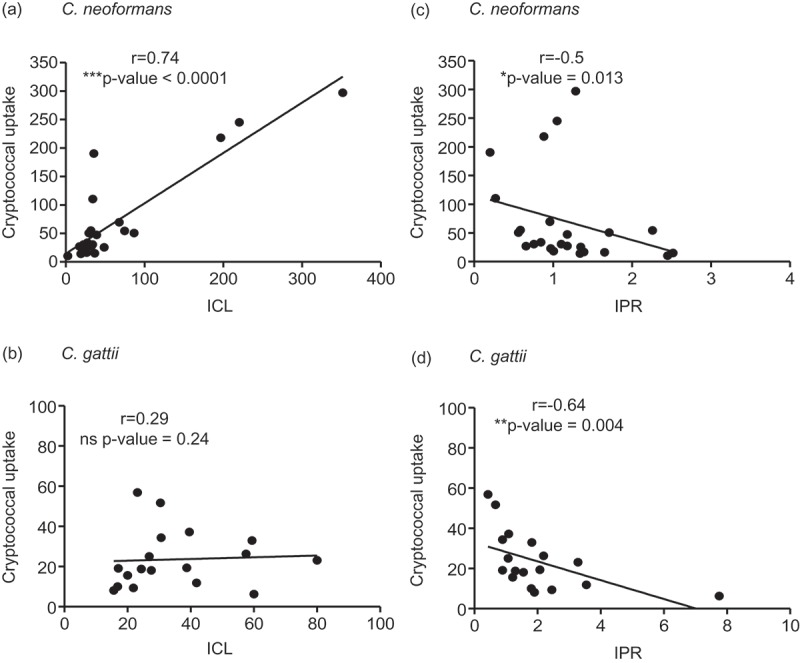
Association of the cryptococcal uptake of *C. neoformans* and *C. gattii* clinical isolate with intracellular load and proliferation within macrophages. *C. neoformans* and *C. gattii* clinical isolates were independently exposed to J774 macrophages and followed by determining the uptake rate, intracellular loads (ICL) and intracellular proliferation rate (IPR) at 24 h postinfection. The correlation between uptake rate and ICL of *C. neoformans* (a) or of *C. gattii* (b) clinical isolates was evaluated. The uptake rate of *C. neoformans* (c) or of *C. gattii* (d) was also assessed for the correlation to IPR. Spearman’s rank test was used to determine the correlation between the uptake rate, ICL and IPR. Correlation coefficients (r) and p-values were given for each correlation. * p <0.05, ** p <0.01, *** p <0.001 and ns = not significantly correlated.

The high uptake rate of *C. neoformans* by macrophages was shown to be positively correlated to the intracellular fungal load and inversely associated with the intracellular proliferation rate [15]. We further determined whether these relationships could be applied in clinical isolates of both *C neoformans* and *C. gattii*. Interestingly, we found a profound positive correlation between the fungal uptake and intracellular fungal load at 24 hours of *C. neoformans* (r =0.74, P <0.0001) (Figure 2(a)), but not that of *C. gattii* (r =0.29, P = 0.24) (Figure 2(b)). The relationship between the fungal uptake and intracellular proliferation rate was found to be inversely correlated for both *C. neoformans* (r =−0.5, P = 0.013) (Figure 2(c)) and *C. gattii* (r =−0.64, P = 0.004) (Figure 2(d)).

**Figure 3.**
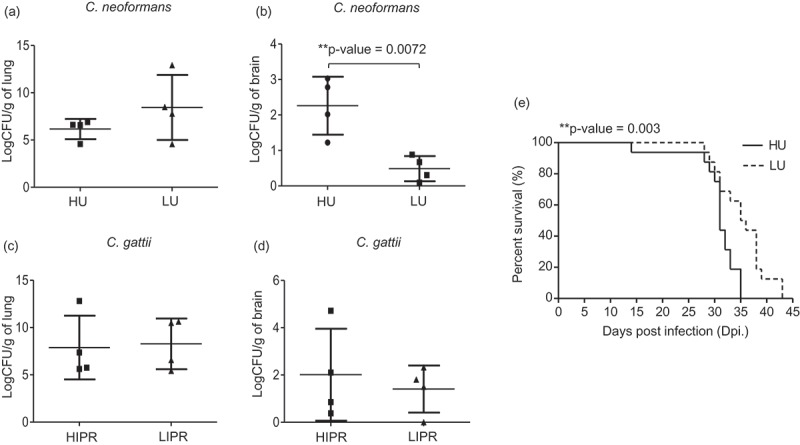
Mice infected with high-uptake *C. neoformans* isolates exhibited higher brain fungal burdens and had reduced survival rate. *C. neoformans* isolates were selected to represent the top 4 high-uptake and bottom 4 low-uptake strains and *C. gattii* isolates were selected to represent the top 4 high IPR and bottom 4 low IPR strains based on J774 macrophage uptake and IPR profiles. (a-d) BALB/c mice were intranasal infected with high- (HU) and low- (LU) uptake *C. neoformans* isolates or high (HIPR) and low IPR (LIPR) *C. gattii* isolates. Lung and brain fungal burdens of mice infected with *C. neoformans* (a, b) and *C. gattii* (c, d) were enumerated at 14 d postinfection. Graphs depict mean ± SD and are representative of 3 experiments with 3 to 5 mice per group. Significance was determined using unpaired t test (2-tailed) ** p <0.01, ns = not significantly different. (e) For survival analysis, BALB/c mice were infected with the high- (HU, solid line) or low- (LU, dot line) uptake strain of *C. neoformans* and were monitored for their survival daily for 60 d. Survival curves were generated from the results obtained with 8 mice per strain and evaluated for statistical significance with Kaplan-Meier survival curves, and P values were obtained from a log-rank test, ** p <0.01.

Previously, the fungal uptake of *C. neoformans* was found to be correlated with the clinical severity of cryptococcal disease in humans [15]. Because we found that *C. neoformans* had greater ability than *C. gattii* to be engulfed by macrophages and the fungal uptake rate was strongly associated with the intracellular fungal load in macrophages, we next tested whether the fungal uptake rate of *C. neoformans* could predict the level of the organ fungal burdens. We selected 4 strains of *C. neoformans* with a high phagocytosis rate (approximately 200 cryptococci/µl lysate) and 4 strains of those with a low phagocytosis rate (<50 cryptococci/µl lysate) to evaluate the lung and brain fungal burdens in a murine cryptococcosis model (Supplementary table 1). Although mice infected with high and low fungal uptake isolates of *C. neoformans* exhibited comparable lung fungal burdens (Figure 3(a)), mice infected with high-uptake *C. neoformans* showed significantly higher brain fungal burdens than those infected with low-uptake strains (Figure 3(b)). Because the intracellular proliferation rate of *C. gattii* was higher than that of *C. neoformans*, we speculated that the high IPR might correlate to lung fungal burdens. Indeed, we did not detect any differences in the lung (Figure 3(c)) or brain (Figure 3(d)) fungal burdens of mice infected *C. gattii* isolates with high IPR (approximately 3) and low IPR (<1) (Supplementary table 1). Because high-uptake *C. neoformans* showed higher tendency to dissemination to the brain, we further investigated mouse survival following infection with those high and low fungal uptake isolates of *C. neoformans*. Consistent with our fungal burdens findings, we found that mice infected with high-uptake *C. neoformans* had significantly reduced survival compared with those infected with low-uptake strains (Figure 3(e), P = 0.003). Altogether, these data emphasize the roles of high fungal uptake in macrophages associated with the ability of *C. neoformans* to disseminate to the brain in promoting disease progression.

### Infection with high-uptake *C. neoforman*s potentiated the induction of alternatively activated macrophages

**Figure 4.**
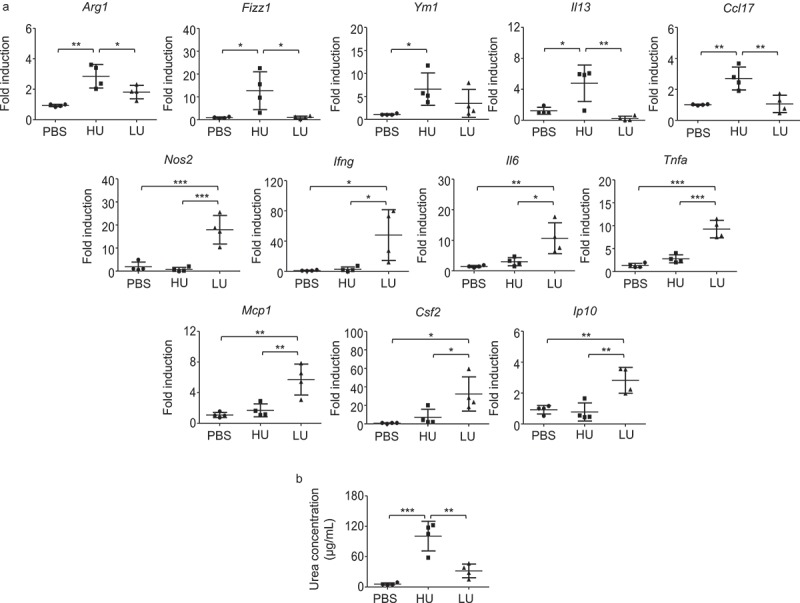
Infection with high-uptake *C. neoformans* strains induced alveolar macrophages with higher expression of genes related to M2 macrophage. BALB/c mice received PBS or were infected with the top 4 high-and the top 4 low-uptake *C. neoformans* isolates. (a) Alveolar macrophages were isolated from the bronchoalveolar lavage of each mouse at 14 d postinfection and total RNA was isolated and subjected to cDNA synthesis, followed by real-time PCR analysis of gene for M1 and M2 including *Arg1, Fizz1, Ym1, Il13, Ccl17, Nos2, Ifng, Il6, Tnfa, Mcp1, Csf2 and Ip10*. Data were expressed as fold induction over actin (*Actb*) expression, with the mRNA levels in the PBS control set as 1. (b) Bronchoalveolar lavages of PBS-treated mice and high-uptake and low-uptake *C. neoformans*-infected mice were collected and the arginase enzyme activity was determined by photometric measurement of produced urea concentration. Graphs depict mean ± SD and are representative of 3 experiments with 3 to 5 mice per group. Significance was determined by one-way ANOVA with Tukey’s post hoc analysis * p <0.05, ** p <0.01, ***p <0.001.

The polarization of macrophages to classical (M1) and alternatively (M2) activated macrophages plays a role in controlling and exacerbating cryptococcal diseases, respectively [20,21]. Although M2 macrophages have been shown to possess high phagocytosis capacity, they promote fungal persistence rather than contribute to fungal clearance [22]. The high uptake rate of *C. neoformans* coupled with the enhanced intracellular loads in macrophages and fungal dissemination to the brain might be associated with the induction of M2 macrophages. To test this possibility, we examined the phenotypes of alveolar macrophages isolated from mice infected with high- and low-uptake *C. neoformans* as described in the above experiment. Mice were infected with high- and low-uptake *C. neoformans* and alveolar macrophages were isolated from the bronchoalveolar lavage following 14 d postinfection. Compared with alveolar macrophages of PBS-treated mice and low-uptake *C. neoformans*-infected mice, those of high-uptake *C. neoformans*-infected mice showed significantly increased expression of genes associated with M2 macrophage, including *Arg1, Fizz1, Il13 and Ccl17* (Figure 4(a)). Interestingly, we observed only the low-but not high-uptake *C. neoformans* induced significantly higher levels of genes related to M1 markers and inflammatory cytokines and chemokines, including *Nos2, Ifng, Il6, Tnfa, Mcp1, Csf2* and *Ip10* (Figure 4(a)). Because arginase 1 is the prototypic marker for M2 macrophages and alveolar macrophages of high-uptake *C. neoformans* infected mice showed significantly higher *Arg1* mRNA expression than those infected with low-uptake strains, we further compared the arginase enzyme activity between high-and low-uptake strains. The arginase enzyme activity of high-uptake strains in BAL fluids was significantly higher than those of low-uptake strains (Figure 4(b)). These data suggested that infection with low-and high-uptake *C. neoformans* results in altered macrophage phenotypes. While low-uptake *C. neoformans* macrophages may drive more inflammatory macrophages, high-uptake *C. neoformans* potentiates the induction of M2-like macrophages.

### *C. neoforman*s strains with high uptake rate promotes type 2 immune responses

**Figure 5.**
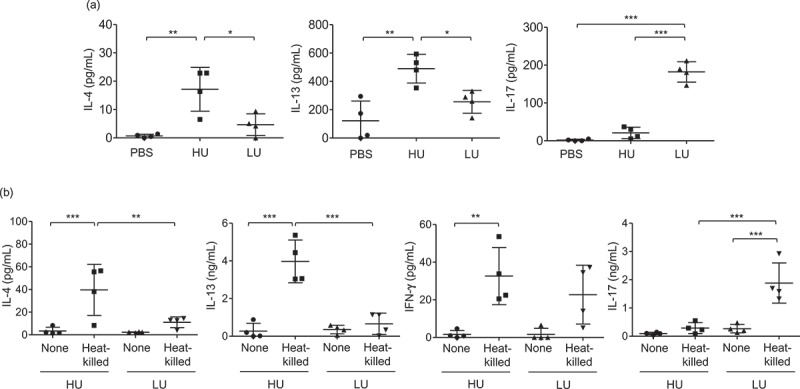
High-uptake *C. neoformans* potentiated the induction of type 2 immune responses and reduced type-17 immune responses in the lungs. BALB/c mice received PBS or were infected with the top 4 high-and the top 4 low-uptake *C. neoformans* isolates. (a) The bronchoalveolar lavages were collected and assessed for cytokine production at 14 d postinfection. (b) Lung draining lymph nodes were harvested and cell suspensions were prepared to analyze antigen-specific cytokine production. After lymph node cells were stimulated with heat-killed *Cryptococcus* cells for 72 h, supernatant was collected and subjected to analyze cytokines by ELISA. Graphs depict mean ± SD and are representative of 3 experiments with 3 to 5 mice per group. Significance was determined by one-way ANOVA with Tukey’s post hoc analysis * p <0.05, ** p <0.01 and *** p <0.001.

While type-1 immune responses have been shown to drive protective immunity against cryptococcal infection, type 2-skewed immune responses defined by the production of IL-4, IL-5, and IL-13 play deleterious roles in infection with *C. neoformans* [23]. Moreover, type-2 cytokine environment has been known to promote the induction of alternative activation of macrophages [24].We further investigated whether the high-uptake *C. neoformans* strain that induced greater disease severity might be involved in inducing type-2 biased immune responses. Mice were infected with high-and low-uptake *C. neoformans* strains as described above and the bronchoalveolar lavage (BAL) cytokines after 14 d post infection were evaluated. We did not detect IFN-γ secretion in the BAL fluids of mice infected with these clinical isolates; however, significantly increased levels of Th2 cytokine IL-4 and IL-13 and Th17 cytokine IL-17 were revealed (Figure 5(a)). Interestingly, the production of IL-4 and IL-13 in mice infected with high-uptake isolates were potently elevated more than those infected with low-uptake isolates (Figure 5(a)). In contrast, infection with low-uptake isolates induced a significantly greater extent of IL-17 cytokine than those infected with high-uptake isolates (P < 0.001) (Figure 5(a)). To further investigate whether the high-and low-phagocytosis rate of *C. neoformans* by macrophages may influence T helper cell differentiation, we next examined the antigen-specific cytokine responses by draining lymph node cells of mice infected with high-and low-uptake isolates co-pulsed with heat-killed *Cryptococcus* cells (Figure 5(b)). Although we did not detect the significant difference of antigen-specific IFN-γ production in the draining lymph node cells of mice infected high and low-uptake infection, the high-uptake-infected groups showed a significantly higher cryptococcal antigen-specific IL-4 and IL-13 production and lower IL-17 production than low uptake-infected groups (Figure 5(b)). We also found the consistent data of antigen-specific cytokine production in the draining lymph node cells stimulated with live *Cryptococcus* cells (Supplementary Figure 1). Altogether, these data indicate that *C. neoformans* with high uptake rate potentiate the induction of type-2 immune responses and the activation of alternative macrophage. These changes in host immune responses driven by high uptake rate *C. neoformans* may promote the enhanced intracellular load in macrophages that contributes to the CNS fungal dissemination and disease progression.

## Discussion

The contribution of macrophage uptake and intracellular proliferation rate in the pathogenesis of *Cryptococcus* infection indicate the influence of early host-pathogen interaction in disease development. Although *C. neoformans* and *C. gattii* infection were shown to have pathological differences, the effect of macrophage interaction with these two pathogenic species has not been clearly investigated. Moreover, the influence of macrophage uptake in modulating phenotypes of macrophages and immune responses has not yet been studied. In the present study, we examined the fungal uptake and intracellular proliferation rate of *C. neoformans* and *C. gattii* isolates. Infection with *C. neoformans* showed a higher fungal uptake but lower intracellular proliferation rate than infection with *C. gattii. C. gattii* strains exhibiting high intracellular proliferation rates caused infection with comparable organ burdens, while *C. neoformans* strains exhibiting high-uptake resulted in infection with higher brain fungal burden. Moreover, infection with the high-uptake strains of *C. neoformans* induced distinct alveolar macrophages expressing higher M2 marker and potentiated type-2 immune response. Thus, our findings revealed different interactions between macrophages and *C. neoformans* and *C. gattii* and how higher uptake strains of *C. neoformans* might affect host immune responses that promote the fungal dissemination and disease progression.

While high-uptake *C. neoformans* was known to be associated with patient CSF fungal burdens and the hypervirulent *C. gattii* strains that exhibited enhanced intracellular proliferation rate was correlated with mouse survival [15,16], the differences of macrophage uptake and proliferation between infections with these two species are less clear. Indeed, we detected that *C. neoformans* strains had higher macrophage uptake rates than *C. gattii* strains. In contrast, the intracellular proliferation rate describing as the ratio of intracellular cryptococci at 24 hours and the number of intracellular cryptococci at 2 hours postinfection was higher in macrophages infected with *C. gattii* than *C. neoformans. C. gattii* exhibiting lower uptake ability than *C. neoformans* is likely because of having a higher capsule size. One related study showed that *C. gattii* capsules were larger than those of *C. neoformans* [25]. Moreover, macrophages were shown to preferentially phagocytose *C. neoformans* strains that had smaller capsules [26]. In contrast to *C. gattii*, our data showed a strong association between the uptake and intracellular loads of *C. neoformans*. Mice infected with high-uptake *C. neoformans* strains showed higher brain fungal burdens that associated with the reduced survival compared with those infected with low-uptake strains. Consistent with a related study [15], the high-uptake *C. neoformans* isolates may be more capable of retaining and surviving inside macrophages. Unlike *C. neoformans*, we did not detect the differences in lung and brain fungal burdens in *C. gattii* with high-and low-intracellular proliferation rates. Previous study suggested that the intracellular proliferation rate of *C. gattii* can predict the survival rate of mice infected with *C. gattii*. Quite possibly, organ fungal burdens may not reflect the survival rate of *C. gattii* infection. Recent study using bone marrow-derived macrophage have suggested that the efficacy of phagocytosis of reference strains of *C. neoformans* (H99) and *C. gattii* (R265, WM161, WM179) may be qualitatively similar [27]. Larger studies including strains from other geographical regions with different types of macrophage should be carried out to support our findings. Moreover, further studies on detailed characterization of other virulent factors between these two species and the their associations with macrophage uptake and intracellular proliferation rates may provide further understanding on the underlying mechanisms involved in the role of macrophage for the pathogenesis of *C. neoformans* and *C. gattii* infection.

The interaction with macrophages is a critical step in the development of cryptococcal pathogenesis. Indeed, macrophages can polarize to classical (M1) or alternatively (M2) activated macrophages, depending on the cytokine environment. Related studies have indicated that M1 macrophage stimulated by IFN-γ is involved in the fungal clearance; however, M2 macrophages polarized by IL-4 promoted fungal proliferation [12,20]. *C. neoformans*, with its high uptake rate may modulate macrophages to the phenotypes that support the fungal retention within cells. Indeed, the alveolar macrophages isolated from mice infected with high-uptake strains exhibited the phenotype of M2-like macrophages by revealing the distinct *Arg1, Fizz1, Ym1* and *Il13* gene expressions when compared with those infected with low-uptake strains. The expressions of Arg1, Fizz1 and IL-13 are usually related to the alternative activation of macrophages. Fizz1 was known to be highly induced in M2 macrophages by IL-4 and IL-13 [28]. IL-13 was known to be expressed by not only Th2 cells but also human M2 macrophages among patients with pulmonary fibrosis as well as in mouse alveolar macrophages exposed to instilled particles [29,30]. Moreover, we observed a higher arginase enzyme activity in bronchoalveolar lavages of mice infected with high-uptake strains. Altogether, these data indicate that infection with high-uptake *C. neoformans* may promote the activation of alternatively activated macrophages that could enhance the retention of cryptococci within macrophage cells.

The type-2 immune response is known to induce the activation of M2 macrophages. Compared with infections with low-uptake strains that secreted higher IL-17, infection with high-uptake *C. neoformans* strains induced higher secretions of bronchoalveolar lavage IL-4 and IL-13, the major cytokine profiles of type-2 immune responses. While the induction of type-1 and type-17 cytokine has been shown to promote the protective immunity against *C. neoformans*, cytokines related to type-2 immunity have been known to promote the persistence and progression of *C. neoformans* infection [8,31–34].

In conclusion, we provided the evidence that cryptococcal-phagocyte interactions that play important roles in mediating disease pathogenesis may modulate types of macrophages and immune responses. *C. neoformans* and *C. gattii* exhibited different capacities in macrophage phagocytosis, fungal retention and proliferation. The high-uptake strains of *C. neoformans* promoted the induction of M2-like macrophages, enhanced arginase enzyme activity and elevated levels of type-2 cytokines that may support the progression of cryptococcal diseases and brain dissemination. Future studies on more clinical isolates of *C. neoformans* and *C. gattii* interaction with macrophages will be instrumental for the understanding of the complex interactions between cryptococci and host macrophages that contribute to cryptococcal diseases.
