# Supplementary material for: Cryptococcus neoformans and Cryptococcus gattii clinical isolates from Thailand display diverse phenotypic interactions with macrophages
Source: Virulence. 2018 Dec 6;10(1):26–36. doi: 10.1080/21505594.2018.1556150 (PMC6298761; doi:10.1080/21505594.2018.1556150)
Supplement: Supplemental Material [file kvir-10-01-1556150-s001.docx]

**Supplementary Table 1. Cryptococcal uptake and intracellular proliferation rate (IPR) at 24 hour-infection of *C. neoformans* and *C. gattii* clinical isolates.**

All clinical isolates of *Cryptococcus* spp. (23 isolates of *C. neoformans* and 18 isolates of *C. gattii*) were evaluated for cryptococcal uptake and IPR at 24 h using J774 macrophages. The 4 high-uptake strains (HU, black box) and 4 low-uptake strains (LU, white box) of *C. neoformans* and the 4 high IPR (HIPR, black circle) and 4 low IPR (LIPR, white circle) of *C. gattii* were selected for further analyses.


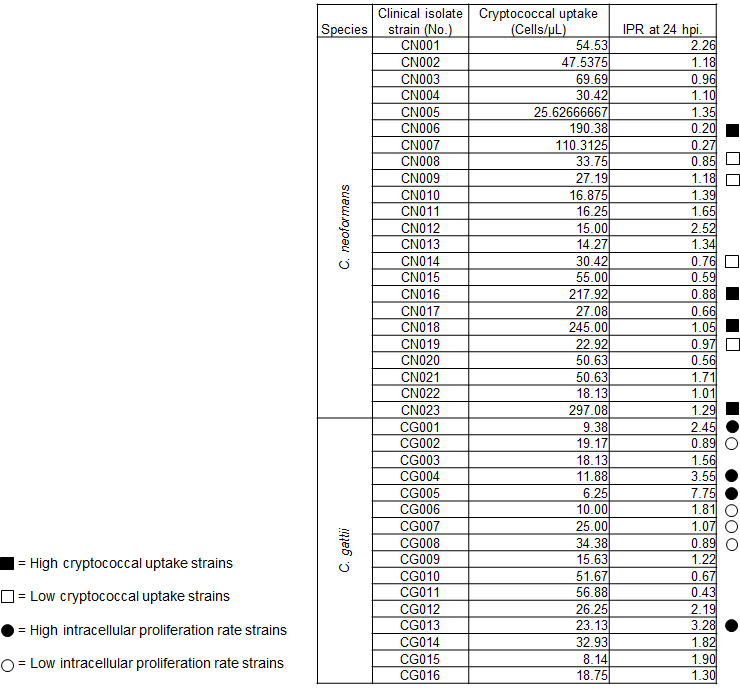


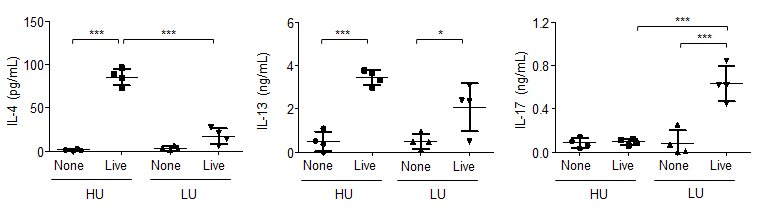


**Supplementary Figure 1. Antigen-specific cytokine responses of lung-draining lymph node cells stimulated with live *Cryptococcus*.**

Lung draining lymph node cells prepared from mice infected with the high- (HU) and low- (LU) uptake strains of *C. neoformans* were plated in a 24-well plate and stimulated with live *Cryptococcus* (at a ratio of 2:1 *Cryptococcus* cells to leukocytes). Following 72 hour stimulation at 37°C with 5% CO_2_, the culture supernatant was collected and kept at -80°C before analysis of cytokine by ELISA * p <0.05, and *** p <0.001.
